# From Traditional to Eco‐Friendly Dentistry: Assessing the Promising Sustainability Outlook in Saudi Arabia

**DOI:** 10.1155/ijod/9714864

**Published:** 2026-06-23

**Authors:** Sanjida Haque, Rosanna Cavalletto, Mohammed Ali Alomshani

**Affiliations:** ^1^ Center for Sustainability and Climate; Center of Excellence in Cybersecurity, Prince Sultan University, Riyadh, Saudi Arabia, psu.edu.sa; ^2^ Environmental and Geological Solutions, Sensing Nature, Riyadh, Saudi Arabia; ^3^ Department of Geology and Geophysics, College of Science, King Saud University, Riyadh, Saudi Arabia, ksu.edu.sa

**Keywords:** amalgam, barriers, dental sustainability, eco-friendly dental practice, green practices, Saudi Arabia

## Abstract

**Background:**

Dentistry is a resource‐hungry segment that produces a lot of waste and also consumes more energy and water. Transitioning toward environmentally sustainable dental practices is essential for reducing the environmental burden of dental services in Saudi Arabia, but there is limited evidence on the subject.

**Purpose:**

The purpose of the study was to explore the knowledge of eco‐friendly dental practices, their existing adoption, and the barriers to the adoption of dental practitioners and clinic directors in Saudi Arabia.

**Methods:**

The study employed a cross‐sectional mixed‐method design. It comprised the quantitative stage, which included a structured questionnaire of 200 dentists or clinic directors working in dental clinics in Riyadh, through six domains: medical devices and equipment, dental amalgam, imaging, paper, energy, and water management. The qualitative step involved six semistructured interviews with key stakeholders in various parts of Saudi Arabia, which were analyzed on the premise of a thematic approach as developed by Braun and Clarke. Two phases were combined to provide a more comprehensive picture of the adoption patterns and the potential impediments.

**Findings:**

Digital imaging, energy efficiency, and paper management had high adoption rates. By contrast, lower implementation was found among reusable syringes, cups, protective packaging, sensor water taps, and renewable energy sources, although the awareness of implementation was high. The most prominent barriers included insufficient training (*M* = 3.96), insufficient motivation (*M* = 3.80), and cost and logistical issues.

**Conclusion:**

Saudi dental clinics appear to be progressing toward sustainability, but specific training programs, monetary incentives, and institutional policy are the key elements that are needed to address the gap between awareness and the application of environmentally friendly operations. The results bring priority areas to the forefront and provide practical learning inquiries to policymakers and professional organizations in the dentistry profession.

## 1. Introduction

Healthcare is one of the world’s most resource‐intensive sectors and plays a notable role in global greenhouse gas emissions [1]. Hospitals and clinics rely heavily on water, energy, single‐use materials, and chemical products, all of which add to the environmental strain [2, 3]. As more and more people around the world pay attention to climate change, the healthcare industry is coming under increasing scrutiny. People are asking the industry to lower its environmental impact while still providing high‐quality care to patients [4]. Dentistry, though smaller in scale compared to that in hospitals, is no exception.

Dental practice requires extensive use of consumables and equipment, ranging from disposable plastics to sterilization products and radiographic materials [5, 6]. These activities generate solid waste, chemical residues, and elevated energy and water consumption. Even common procedures, such as radiography, can produce significant chemical effluents if conventional film is used. Amalgam disposal, meanwhile, has long been recognized as a source of mercury release into the environment [7]; it is a hidden source of global mercury pollution [8]. About 8% of the dentists get rid of the excess silver amalgam in a common bin (nonmedical waste) [9], and 50% of mercury from dental amalgams is entering the wastewater treatment plants; its levels in the sewer system from 330 dental operators in Saudi Arabia exceed the maximum allowance of 50 μg/L [10]. Collectively, these factors illustrate why dentistry is considered a high‐impact branch of healthcare when examined through the lens of sustainability.

To counter this, the term “green dentistry” has been coined. The method supports the integration of preventive, restorative, and surgical therapy by practitioners alongside activities that can be targeted at lowering environmental degradation. Sustainability can be achieved in dentistry through the minimization of the ethical footprint in terms of the environment [11, 12]. Such transitions might include moving to the digitization of radiography in the case of a film‐to‐digital transition, the use of electronic records instead of paper records, reusable tool use where the consequences of such a move are safe, and water and energy conservation. Such efforts are of added value to environmental protection. They are also capable of reducing operating expenses, improving patient confidence, and making the name of dental care closer to broader healthcare sustainability goals.

In Saudi Arabia, the Vision 2030 and the Saudi Green Initiative are among the national strategies that indicate that the government is determined to pursue an ecologically sound development [13]. While advancements have occurred in areas like energy and transport, research has largely overlooked dentistry. The new research shed light on the existing obstacles. Dentists in Riyadh are aware of the significance of sustainability but lack access to funds and relevant sources of green materials, as well as insufficient knowledge on sustainability due to a lack of adequate training [14]. Dental students in their studies are currently aware of the sustainability objectives, yet they usually do not receive practical guidance regarding the implementation of these objectives in their regular practice [15].

This gap highlights the need for further research. This research paper will analyze the meaning of sustainability for dental professionals in Saudi Arabia, current dental practices that are already green, and barriers to implementing greener dental practices. The combination of survey and qualitative knowledge in the study will help provide evidence that will guide the professional training, development of policies, and overall conformity of dental practice to national sustainability strategies.

## 2. Material and Methods

### 2.1. Study Design

This research paper used a cross‐sectional, mixed‐method design to investigate the awareness, adoption, and barriers of sustainable dentistry practices in Saudi Arabia. The researchers intentionally combined a quantitative survey (to identify general, measurable patterns in 200 clinics) with semistructured interviews (to provide insights into those patterns). Qualitative results played a complementary and explanatory role—these were helpful in explaining the patterns and anomalies found in the data gathered through the survey, especially where high awareness did not equate to adoption. This sequential explanatory integration enhanced the trustworthiness of the findings by allowing qualitative insights to contextualize quantitative patterns [16].

The study was ethically approved by the Prince Sultan University Institutional Review Board (PSU IRB‐2023‐03‐0145).

### 2.2. Quantitative Phase

Data were collected with an online structured questionnaire for more than 6 months (June 2024–2 December 2024). The instrument was based on past validated studies in the research of sustainable dental practices [17, 18]. The research team used the review of the adapted items in terms of conceptual agreeability, clarity, and applicability to situations found in the Saudi dental context prior to the data collection process. Ten dental practitioners not so included in the main sample were then piloted with the questionnaire to determine its clarity and face validity; some wording changes were then made based on pilot feedback. The internal consistency of items related to barriers was measured using Cronbach alpha = 0.78, which is acceptable.

The respondents were 200 dentists or clinic directors in 200 Riyadh dental clinics in Saudi Arabia. Riyadh was chosen as the site of study due to its location as the national capital, being the most populated and a commercial hub in Saudi Arabia, and the large number of dental clinics, which makes it a well‐placed representative city. The survey included sociodemographic data and assessed implementation of sustainable practices in six domains: medical equipment and medical devices, dental amalgam, imaging, paper usage, energy, and water management. The process was anonymous and conducted through Google Forms, with participants allowed to fill out one form only. All the participants were made aware of the aim of the research, and informed consent was collected prior to the study.

To determine and classify the barriers, a four‐point Likert scale was used (1 = not a barrier and 4 = significant barrier). Although the data were ordinal in nature due to their derivation from Likert‐scale responses, one‐way ANOVA was used on an exploratory basis, taking into account the sample size (*n* = 200) and the objectives of studying broad group‐level variation within each demographic category, which aligns with the accepted tradition in health‐profession surveys. The reported results include the precise *F*‐statistics as well as *p*‐values. Since the analysis is exploratory, when needed, effect sizes (partial *η* ^2^) are provided, and post hoc comparisons (Tukey HSD) are performed, where overall ANOVA results were found to be significant. All the analyses have been done in SPSS version 25 with the significance level cutoff at *p*  < 0.05.

### 2.3. Qualitative Phase

Key stakeholders such as dentists, dental hygienists, clinic owners, and administrators in various parts of Saudi Arabia were interviewed in six semistructured interviews. The qualitative aspect dictated the choice of six interviews due to the theoretical aim of the latter, which was to shed light on the contextual drivers and barriers established in a quantitative manner instead of pursuing independent thematic saturation. The method is likely to be connected to purposive sampling within mixed‐method studies in that the qualitative portion will complement, rather than substitute, the evidence provided in the survey [16]. Interviews were, however, continued until no new themes of significance came out among participants, and it is reasonable to say that they will be adequate in terms of the explanatory purpose intended.

They engaged in discussions on day‐to‐day sustainability practices, incentives, perceived obstacles, and opinions about policy support. All interviews were tape‐recorded under the consent of the participants, transcribed verbatim, and organized into themes according to a six‐phase framework suggested by Braun and Clarke. Two researchers in the research team generated initial codes, and then, subsequently, themes were identified, named, and refined through joint reviewing. Coding discrepancies were resolved through discussion and consensus among the research team. This process was carried out in the strictest terms of confidentiality and anonymity.

## 3. Results

### 3.1. Participants Demographic Characteristics

The survey included dentists or clinic directors from 200 dental clinics, comprising 154 males (77%) and 46 females (23%). Most participants were aged 30–39 years (44.5%), followed by those aged 40–49 years (30%). Regarding professional experience, nearly half had 11–15 years (48%), with smaller proportions reporting ≤10 years (16%), 16–25 years (21.5%), 26–35 years (11.5%), and >35 years (3%). Table 1 provides the demographic characteristics of survey participants.

**Table 1:** Demographic characteristics of survey participants (*n* = 200).

Variable	*n*	%
Gender		
Female	46	23.0
Male	154	77.0
Age group (years)		
20–29	15	7.5
30–39	89	44.5
40–49	60	30.0
50–59	30	15.0
≥60	6	3.0
Years of experience		
≤10	32	16.0
11–15	96	48.0
16–25	43	21.5
26–35	23	11.5
>35	6	3.0

### 3.2. Adoption of Eco‐Friendly Dental Practices

The adoption of eco‐friendly dental practices varied significantly across the six analyzed areas in dental clinics in Riyadh, Saudi Arabia. The adoption rates of medical devices and equipment were the greatest with metal trays (100%) and reusable bib holders (92%), as well as with digital scanning commonly practiced (81%). Nevertheless, reusable cups (2.5%), reusable syringes (3.5%), and reusable protective packing (2.5%) were adopted in small numbers. A significant percentage of participants claimed they knew but did not apply separate sterilization pouches (65%) and protective packaging (50%), which means that there is a large knowledge‐protection gap between familiarity and use. The dental amalgam management demonstrated the universal use of alternative restorative materials (100%) and the nonuse of plumbing disposal (90.5%). Average airtight amalgam storage (51.5% use) was in marked contrast, affected by low take‐up of sediment amalgam separators (10.5%), despite 51% awareness, indicating obstacles in knowledge to action (Table 2).

**Table 2:** Implementation status of eco‐friendly practices across domains (*n* = 200).

Domain	Implemented (%)	In progress (%)	Not implemented (%)	Aware but not implemented (%)
Medical devices/equipment	29.8	1.7	44.1	25.9
Amalgam management	68.2	0	10.5	20.8
Imaging management	95	0	0	5
Paper management	70.4	0	9.2	20.6
Energy management	86.3`	0	0.8	12.9
Water management	84.9	3.5	10.5	1.1

Imaging management showed almost universal use with digital imaging (99%); safe chemical handling (93%); and recycling of liquids, largely used, and only a few responses with awareness only (<7%). Practices related to paper management were extensively used in the areas of digital patient management (96.5%) and essential/double‐sided printing (100%) and diminished in use in those connected to recyclable paper (18.5%) and donating old dental books (3.5%), with high awareness and limited usage. Energy Management had nearly universal applications in terms of LEDs, motion‐sensor lights, and the use of energy‐efficient appliances, but the use of renewable energy has been notably low (7.5%), even with 87% awareness. Water Management indicated high use of hand sanitizer (100%) and dual‐flush toilets (96%); sensor taps were used moderately (45%), and 14% of the clinics were experimenting with their use (Table 2).

### 3.3. Barriers to Implementing Eco‐Friendly Dental Practices

Barriers to implementing eco‐friendly dental practices were identified in the survey and ranked using a 1–4 Likert scale. The most notable challenges were lack of training (*M* = 3.96) and lack of motivation (*M* = 3.80), which indicated internal and organizational barriers. Costs, human resources, and logistics had moderate barriers (*M* = 3.41–3.56). The most insignificant barriers were the availability of materials (*M* = 2.00) and the awareness of patients (*M* = 2.24), which suggests that the workforce capacity and incentives are the main factors in implementation (Table 3).

**Table 3:** Mean scores and ranking of barriers to implementation of eco‐friendly dental practices (*n* = 200).

Barrier	Mean	SD	Rank
Lack of training	3.96	0.20	1
Lack of motivation	3.80	0.40	2
Costs	3.56	0.52	3
Human resources	3.46	0.57	4
Logistics	3.41	0.49	5
Operationalization	3.15	0.42	6
Implementation time	2.48	0.61	7
Patients’ awareness	2.24	0.68	8
Material availability	2.00	0.41	9

One‐way ANOVA was done to determine if there was an association between gender, age bracket, and years of professional experience with adoption in the six domains. For medical devices and equipment, no statistically significant differences were found by gender (*F* [1198] = 0.84, *p* = 0.361, *η* ^2^ = 0.004), age group (*F* [4195] = 1.12, *p* = 0.348, *η* ^2^ = 0.022), or years of experience (*F* [4195] = 0.97, *p* = 0.424, *η* ^2^ = 0.019). Likewise, for dental amalgam management and, indeed, image management, there was no significant difference in adoption by any demographic variable (all *p*  > 0.05), and this result indicates that adoption of these areas is largely informed by the norms of the institution, equipment availability, and standardized clinical protocols and not individual characteristics (Table 4).

**Table 4:** One‐way ANOVA results for demographic differences in eco‐friendly dental practice adoption.

Domain	Variable	*F*	*df*	*p*‐Value	*η* ^2^	Post hoc (Tukey’s HSD)
Medical devices and equipment	Gender	0.84	1198	0.361	0.004	N/A
	Age	1.12	4195	0.348	0.022	No significant pairs
	Y/E^a^	0.97	4195	0.424	0.019	No significant pairs
Dental amalgam management	Gender	0.76	1198	0.384	0.004	N/A
	Age	1.03	4195	0.392	0.021	No significant pairs
	Y/E	0.88	4195	0.477	0.018	No significant pairs
Imaging management	Gender	0.61	1198	0.436	0.003	N/A
	Age	0.94	4195	0.441	0.019	No significant pairs
	Y/E	0.79	4195	0.533	0.016	No significant pairs
Paper management	Gender	1.14	1198	0.287	0.006	N/A
	Age	2.08	4195	0.084	0.041	No significant pairs
	Y/E	3.21	4195	0.014^a^	0.062	16–25 years < 11–15 years (*p* = 0.031)
Energy management	Gender	0.92	1198	0.339	0.005	N/A
	Age	1.44	4195	0.221	0.029	No significant pairs
	Y/E	1.31	4195	0.267	0.026	No significant pairs
Water management	Gender	1.08	1198	0.300	0.005	N/A
	Age	3.47	4195	0.009^a^	0.066	50–59 years < 30–39 years (*p* = 0.018)
	Y/E	2.94	4195	0.021^a^	0.057	>15 years < ≤15 years (*p* = 0.024)

^a^Y/E: Years of experience.

Based on years of experience, there was a significant difference in paper management practices (*F* [4195] = 3.21, *p* = 0.014, *η* ^2^ = 0.062), and post hoc analysis (Tukey HSD) revealed that practitioners with 16 years or more of experience correlated with lower adoption of digital documentation compared to those with 1 year of experience. No significant difference was found by age group alone (*F* [4195] = 2.08, *p* = 0.084). The adoption of water management was significantly related to both the age group (*F* [4195] = 3.47, *p* = 0.009, *η* ^2^ = 0.066) and years of experience (*F* [4195] = 2.94, *p* = 0.021, *η* ^2^ = 0.057), particularly among younger practitioners and those with less than 15 years of experience. There was no meaningful difference in energy management by demographic (all *p*  > 0.05), which is in line with the overall rating of LED lighting and energy‐efficient equipment by almost all participants.

On the whole, the findings of the ANOVA indicate that demographic factors can be used to explain most areas to a limited extent. Probably, more robust predictors of adopting the eco‐friendly practice in the Saudi dental clinics are the organizational infrastructure, profession training, and institutional policy (Table 4).

### 3.4. Qualitative Findings

The interviews highlighted several themes that reveal how dental professionals in Saudi Arabia perceive and practice eco‐friendly dentistry. Leadership and responsibility emerged strongly, with many interviewees linking sustainability to the role of clinic directors or department heads. They stressed the need for top–down leadership to integrate change into daily routines. One dentist explained, *“As the head of the Strategy Department, it’s my job to plan for the long term… and make sure our operations are more environmentally friendly”* (Interviewee 1).

Practical awareness was evident in small but consistent steps. Actions such as reducing paper use, switching off unused equipment, and choosing reusable tools were mentioned as achievable starting points. These examples show that sustainability is not abstract but is embedded in daily choices. Another recurring theme was the operational focus on reducing waste and managing costs. Several participants noted that financial efficiency and environmental responsibility often overlap. As one put it, *“Managing the clinic in a way that reduces waste and lowers costs… implementing proper waste segregation, monitoring electricity use”* (Interviewee 3).

Comprehensive responsibility also featured strongly, with participants stressing that sustainability goes beyond isolated measures. It includes energy use, water management, materials, and patient education. Interviewees described adopting digital systems, energy‐efficient equipment, and campaigns to raise awareness among patients. The use of eco‐friendly materials further demonstrated this shift. Dentists reported moving toward digital radiography, minimizing single‐use plastics, and conserving water and energy wherever possible. Such measures were described as both practical and aligned with professional responsibility.

Finally, barriers were discussed openly. The most common hurdles were high costs and staff resistance. Some clinics recognized the long‐term benefits but struggled with upfront investments. Other researchers found it difficult to change established staff habits. As one dentist observed, *“Convincing staff to follow new policies is difficult. People are used to traditional ways of doing things, and changing habits takes time”* (Interviewee 3) (Table 5).

**Table 5:** Thematic analysis of interviews with dental professionals on sustainability practices and perceptions in Saudi Arabia.

Theme	Illustrative quote
Role and responsibility	“As the head of the Strategy Department, it’s my job to plan for the long term… and make sure our operations are more environmentally friendly.” (Interviewee 1)
Practical awareness	“Eco‐friendly dental practice is about integrating sustainability into daily hygiene routines.” (Interviewee 2)
Operational focus on waste and cost reduction	“Managing the clinic in a way that reduces waste and lowers costs… implementing proper waste segregation, monitoring electricity use.” (Interviewee 3)
Comprehensive environmental responsibility	“Every aspect of clinic operation requires intentional decisions… invest in energy‐efficient equipment, digital records, and educating patients about sustainable practices.” (Interviewee 5)
Use of eco‐friendly materials	“Using materials and techniques that are less harmful to the environment… reducing single‐use plastics, minimizing water and energy consumption, using digital X‐rays.” (Interviewee 6)
Barriers: cost and staff resistance	“Convincing staff to follow new policies is difficult. People are used to traditional ways of doing things, and changing habits takes time.” (Interviewee 3)

## 4. Discussion

This study revealed the way in which Saudi dentists are approaching eco‐friendly practice as well as revealed the areas where difficulties persist. There are certain sustainable practices already well integrated into day‐to‐day work. As an example, metal trays are used, reusable bib holders are used, and there is digital scanning. These are practices that are simple to implement and have direct short‐term operational or regulatory payoffs. The findings align with global trends, where organizations adopt time‐saving or workflow‐maximizing measures early on.

Other practices, such as reusable cups, syringes, and protective packaging, remain less common. This highlights a clear gap between awareness and action. Dentists may know about these options, but knowledge alone does not translate into routine use. A dentist explained: *“The cost of implementing green practices is simply too high for a small clinic like mine”* (Interviewee 2). This concern also aligns with the survey data, where cost is ranked among the most significant barriers.

Imaging and paper management were more widely practiced, likely because these areas are backed by digital systems and institutional policies. Digital imaging reduces chemical waste and improves safety, while electronic records cut paper waste and improve efficiency [19, 20]. However, water‐ and energy‐saving measures, such as motion‐sensor taps or renewable energy, were rare. Many dentists reported awareness but noted economic and infrastructure barriers. As one interviewee put it: “*Sometimes we want to use reusable items*, *but patients expect disposables—they see it as cleaner”* (Interviewee 4). This shows that patient perception, in addition to cost, shapes dentists’ decisions.

From a patient care and safety perspective, eco‐friendly practices are not only about the environment but also about reducing harm. Digital imaging lowers patient exposure to chemicals, while safer amalgam disposal protects both staff and community water supplies [21]. This reinforces the value of sustainable practice as part of high‐quality patient care.

These findings have several limitations that impact the generalizability of the findings and need to be taken into account when interpreting the findings. The sample was limited to Riyadh‐based clinics, and most clinics had their practices in privacy. This topographical and industry‐related clustering restricts the extrapolation of the results to the context of further public clinics, rural work, or even dental practices in other Saudi areas that have a wide range of different infrastructures, financial resources, or regulatory conditions. The choice of Riyadh was supported by pragmatic and strategic considerations; it is the largest city and most concentrated area of dental practice in Saudi Arabia, but this implies that even inferences must be limited to urban and mostly private dental practice and not to Saudi dentistry. The additional sampling constituted by more than three regions and sectors in the future would offer a stronger premise regarding national claims.

## 5. Limitations

The given research provides useful information but has several significant limitations. To begin with, self‐administered questionnaires were used to collect data, thus making them vulnerable to social desirability and recall error. There is a possibility of some respondents exaggerating their adoption of environmentally friendly practices; hence, an increase in the rates of implementation will be overestimated. Second, only dental clinics in Riyadh were included in the sample, with most being in a private environment. This limits the generalization of the results to the practices in the public sector, rural clinics, or other locations in Saudi Arabia where resources may be very different and the structures of institutional support are different. Findings should then be interpreted as applicable to an urban and noncommunicable dental practice setting but not to Saudi dentistry in general.

Third, as much as practice‐level adoption patterns were taken care of through the questionnaire, it was not as good at delving deeper into the realms of psychological or systemic restraints that might be underlying the perception of the awareness‐to‐action gap. This was partially achieved through the qualitative interviews, although this is constrained by the small number of participants in the interview (*n* = 6) to provide a depth of qualitative conclusions and to transfer the findings. Fourth, two team members applied the thematic analysis technique, and the intercoder agreement was obtained via the discussion, although the actual value of the reliability coefficient was not calculated, which is considered to be a limitation of qualitative rigor. Fifth, the cross‐sectional design does not allow for evaluating the way the sustainable practices develop. A longitudinal study of the same clinics with multiple time points would provide more substantial data regarding the direction of adoption and how any policy or training interventions that may have been introduced between time points affect this direction. Lastly, although ANOVA was employed to investigate the demographic relationships, the ordinality of Likert‐scale data dictates that the parametric results should be used with due caution, and the effect sizes were relatively small in general.

## 6. Conclusion

The results of the current research indicate that the process of dental professionals shifting towards operating in an ecologically friendly environment in Riyadh, Saudi Arabia, is slow, yet the gradual increase in the scope of the issue can be traced to the aspects of digital imaging, energy conservation, and minimization of paper usage, where acceptance of new changes in patient care is supported by the current infrastructure, institutional policy, and the apparent benefits of the suggested change on the operation. Nevertheless, the utilization of reusable materials, renewable energy, and water conservation techniques remains low, indicating a continued lack of appreciation for the action gap.

The main barriers, including insufficient training, lack of motivation, and high initial expenses, are not only lifted in both quantitative and qualitative data but also suggest the specificity of required interventions rather than their generalization. The most likely measures that can transform awareness into practice changes include structured continuing education, financial incentives on green procurement, and strengthening institutional policies.

A sample of urban dental clinics (mostly private) in Riyadh was used to derive these findings. Without additional evidence, they cannot be generalized to be reflective of all Saudi Arabian dental practices. However, they can serve as a helpful empirical basis for which policymakers and professional associations interested in influencing sustainability policies in the dental industry. Conformance with the visions of Saudi Vision 2030 and the Saudi Green Initiative would offer a framework at the country level within which such measures can be properly located, but it will take time longer than just raising awareness before yielding tangible results.

## Author Contributions

**Sanjida Haque:** conceptualization, methodology, formal analysis, data interpretation, writing – review and editing, supervision. **Rosanna Cavalletto:** conceptualization, methodology, writing – original draft, writing – review and editing. **Mohammed Ali Alomshani:** investigation, data curation, writing – original draft.

## Funding

The authors received no specific funding for this work.

## Disclosure

All the intellectual content, analysis, and interpretations remain the responsibility of the authors.

## Conflicts of Interest

The authors declare no conflicts of interest.

## Data Availability

The data that support the findings of this study are available upon request from the corresponding author. The data are not publicly available due to privacy or ethical restrictions.
